# In my own time: A non–cell-autonomous circadian regulation in plant cells

**DOI:** 10.1093/plphys/kiad303

**Published:** 2023-05-22

**Authors:** José Manuel Ugalde, Aida Maric

**Affiliations:** Assistant Features Editor, Plant Physiology, American Society of Plant Biologists, Rockville, MD, USA; Institute of Crop Science and Resource Conservation (INRES)-Chemical Signalling, University of Bonn, 53113 Bonn, Germany; Assistant Features Editor, Plant Physiology, American Society of Plant Biologists, Rockville, MD, USA; CIBSS-Centre for Integrative Biological Signalling Studies, University of Freiburg, 79104, Freiburg, Germany; Plant Environmental Signalling and Development, Institute of Biology III, University of Freiburg, 79104 Freiburg, Germany

How do plants measure daylength? Why do humans go to sleep at night? Why do roosters “sing” at the same time every day? The answer to these questions lies in the existence of a biological timekeeper innate to most living organisms—the circadian clock (Latin: circa, around; dies, day). The circadian clock allows perception and anticipation of daily and seasonal changes resulting from the earth’s rotation. The molecular mechanisms of circadian clock regulation evolved independently several times, testimony to its importance (Nohales and Kay 2016). In plants, the clock regulation is based on a set of genes with peak expression in either the morning or the evening. These genes regulate each other through transcriptional-translational feedback loops. As examples of the evening-expressed genes, we have a regulation hub partially composed of *LUX ARRHYTHMO* (*LUX*) and *EARLY FLOWERING 3* (*ELF3*). In addition to transcriptional-translational feedback loops, the clock is tightly regulated at multiple levels, including epigenetic, posttranslational, or metabolic control (Maric and Mas 2020; Cervela-Cardona et al. 2021; Yan et al. 2021). This highly sophisticated network regulates a wide range of behavioral and physiological processes in plants (McClung 2019).

It has been established that the behavior of the circadian oscillators is regulated by the cell-autonomous clock gene circuit. Yet not all genes share the same expression period (Nohales 2021). Furthermore, studies in the nuisance plant duckweed (*Lemma minor*) have revealed that cells within the same tissue develop desynchronization of the cell-autonomous clock under different light regimes (Ueno et al. 2022), an idea that has been confirmed using luminescent reporters under the control of clock-related promoters in duckweed (Watanabe et al. 2021). It is not clear whether there is an independent autonomous and non-autonomous rhythm in gene expression and, if so, communication between clocks of different tissues might be regulated by mobile molecules from different tissues.

**Figure 1.**
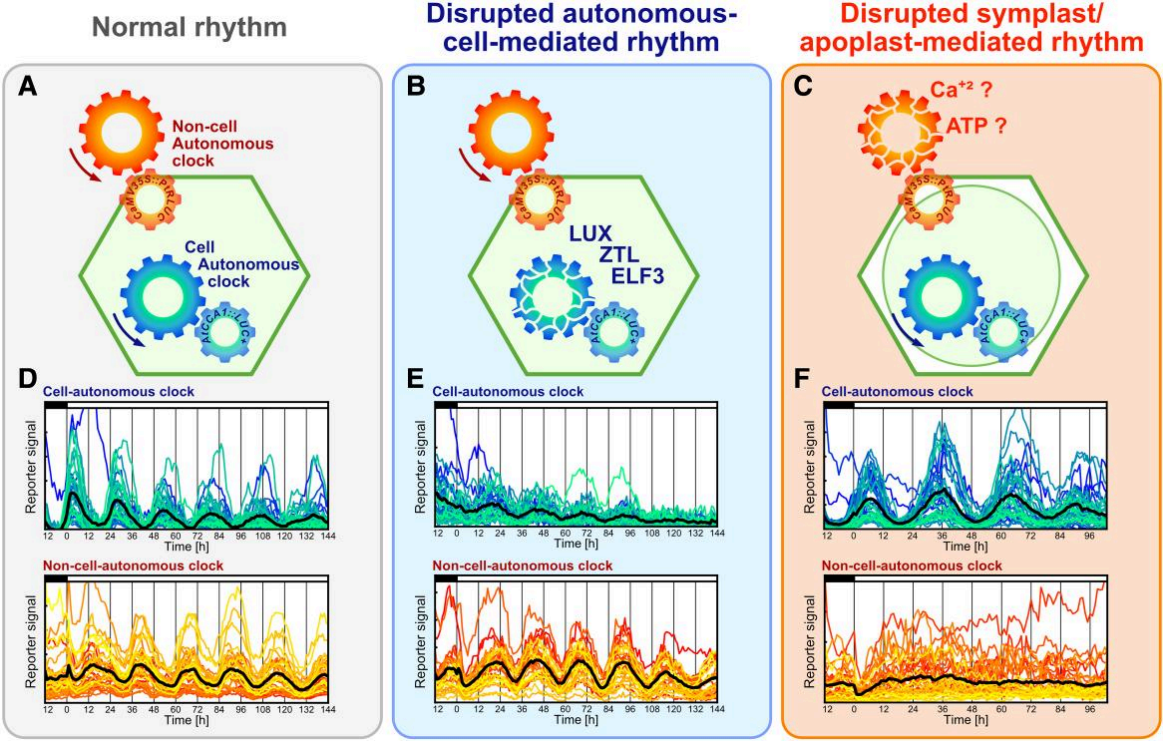
Cell-autonomous and non–cell-autonomous–mediated circadian rhythms in duckweed cells. Coexpression of the bioluminescent reporters *AtCCA1::LUC +* and *CaMV35S::PtRLUC* serves as proxy to follow the cell-autonomous clock (green-blue, bottom gears) or the symplast/apoplast-mediated clock (red-orange, top gears), respectively. Duckweed cells expressing both reporters were maintained unaffected to calibrate oscillation of the reporters (normal rhythm; **A**, **D**), whereas the autonomous cell–mediated rhythm was disrupted by the overexpression of the evening complex genes *LUX*, *ELF3*, or *ZTL* (**B**, **D**). **C**, **F)** To disrupt the non–cell-autonomous clock, duckweed cells were incubated in a plasmolysis-inducing solution. Adapted from Watanabe et al., 2023. Figure created by J.M.U in Affinity designer (Version 2.0.4).

In this issue of *Plant Physiology*, Watanabe et al. 2023 demonstrated that the rhythmic behavior of the luminescent reporter *AtCCA1::LUC +* depends on the autonomous cell clock, whereas a second reporter, *CaMV35S::PtRLUC*, does not. Rather, *CaMV35S::PtRLUC* depends on the communication with the neighboring cells. They initially characterized both transcriptional luminescent reporters, confirming that they are rhythmically coexpressed in duckweed cells and that they maintain such rhythm under free-running periods in constant light (Fig. 1, A and D). To investigate the regulation of these promoters by the autonomous circadian clock, the authors overexpressed clock genes *ZEITLUPE* (*ZTL*), *LUX*, or *ELF3*. They found that overexpression of any of these genes disrupted the rhythm of the *CCA1* reporter but not the *35S* reporter (Fig. 1, B and E). Yet exposure to a plasmolysis-inducing hypertonic medium extended the rhythm of the *CCA1* reporter but shockingly made the rhythm of the *35S* reporter disappear (Fig. 1, C and F). Altogether, these results suggest that the *35S* promoter is not directly regulated by the autonomous clock gene circuit but rather by components of the surrounding tissues.

Watanabe et al. 2023 report the disruption of the autonomous cell signaling by overexpressing the members of the evening complex. It would be very interesting to resolve whether overexpression of morning-expressed clock genes would have the same effect on cell-to-cell communication, excluding that this is a dependent function of the evening complex. Studies in mammals as well as in plants have reported hierarchical relationships between the molecular clocks of tissues distant from each other (Takahashi et al. 2015). This means that somehow in plants, the shoot clock is able to communicate information and regulate the root clock oscillations by deploying a still-unknown motile clock signal (e.g. small proteins) through the plant vasculature (Chen et al. 2020). The communication between clocks allows synchronization of rhythmic response at a plant level. Community-wide efforts to find other non–cell-autonomous elements regulating intercellular communication between clocks has been long underway. Strong candidates include ATP or ions such as calcium (Ca^2+^) due to its fast accumulation and diffusion rates. Fluorescent dynamic biosensors, such as Cameleon or YC-Nano, could give us insight in high resolution of the calcium wave through different cells (Grenzi et al. 2021). Additionally, it will be of great interest to use reporter lines that are not dependent on substrate uptake (e.g. Luciferin). The combination of new biosensors with micrografting can be a strong tool for the identification of new molecules regulating non–cell-autonomous rhythms. This study by Watanabe et al. 2023 opens a window to an exciting niche of research in biological rhythms, which may help to solve the question of what makes the non-autonomous clock go on its own time.
